# Late Auditory evoked potentials in individuals with tinnitus

**DOI:** 10.1590/S1808-86942010000200019

**Published:** 2015-10-19

**Authors:** Valdete Alves Valentins dos Santos Filha, Carla Gentile Matas

**Affiliations:** PhD in Human Communication - Speech and Hearing Therapy Program - Department of Physical Therapy, Speech and Hearing Therapy and Occupational Therapy of the Medical School of the University of São Paulo - FMUSP, Speech and Hearing Therapist; PhD - Federal University of São Paulo - UNIFESP, Speech and Hearing Therapist - Adjunct Professor - Speech and Hearing Therapy Program - Department of Physical Therapy, Speech and Hearing Therapy and Occupational Therapy of the Medical School of the University of São Paulo - FMUSP

**Keywords:** evoked potentials, occupational, noise, tinnitus

## Abstract

Long latency auditory evoked potentials (LLAEP) alterations in individuals with tinnitus are suggestive of dysfunction in the central auditory pathways at a cortical level.

**Aim:**

to characterize the LLAEP in individuals with and without tinnitus exposed to occupational noise.

**Method:**

Cross-sectional contemporary cohort, prospective study. Sixty subjects exposed to occupational noise, ranging in age from 29 to 50 years underwent LLAEP assessment; 30 of them had tinnitus complaint and 30 did not have tinnitus.

**Results:**

we observed significant statistical difference regarding the mean values of latencies of waves N1 (p<0.001), P2 (p=0.002) and P300 (p=0.039) when we compared individuals with and without tinnitus. In individuals with tinnitus we also noticed a greater number of altered results concerning components N1 (60%) and P2 (66.7%), although only component P2 presented significant statistical difference (p=0.010). For the LLAEP, the latency increase was the only type of alteration found (p=1.000). We found a greater association between bilateral tinnitus and bilateral alteration for all components N1(73%), P2(73%) and P300(50%).

**Conclusion:**

It is relevant to study LLAEP in individuals with tinnitus exposed to high occupational sound pressure levels.

## INTRODUCTION

Some people experience a sound sensation which is not related to any external source of stimulation or noise. This auditory phenomenon, defined by the hearing of sounds on one or on both ears, as well as in one's head is known as tinnitus.

Usually, these people perceive tinnitus so strongly that they have impairments in their quality of life, which could impact their work, sleep and communication. Often times, tinnitus exceeds the person's tolerance and adaptive capacity, which may cause physical, mental and/or emotional exhaustion.

Tinnitus is being discussed in writings since the ancient Egyptian civilization. Although present in the lives of people for almost five thousand years, this complaint, especially subjective, remains obscure in many of its aspects, especially regarding its origin^1^.

In an attempt to explain some of the aspects associated with this phenomenon, many authors have raised hypothesis on the possible causes for it. Until the early 80′s, it was believed that tinnitus was a phenomenon which would happen in the cochlea only. Later studies showed that such symptom may involve not only the cochlea, but also the auditory pathways and the cerebral cortex.

Hazell^2^ believes that tinnitus originates in the cochlea and/or in the brainstem as a weak signal, which goes through filtering and amplification before it is perceived at a cortical/subcortical level. This neural activity happens in all of us; however, emotional issues and stress can increase the perception of tinnitus. The perception of such symptom depends on the subcortical filters associated with the processing of signals and of the reaction to tinnitus by the limbic system. Based on real experiences, the individual with such symptom believes that the tinnitus sound is generated in the ear. Nonetheless, from the neurophysiological standpoint, this symptom is described as a perception which happens in the cortical areas reserved for sensorial modes^2^.

Aiming at studying the CNS participation in tinnitus in individuals with and without this symptom, Attias et al. (1993)^3^ carried out a study to check whether central neural activity and/or cognitive processes and/or perception deficits would have an effect on the subjective sensation of tinnitus. The authors used the LLAEP (N1, P2 and P300) and found, in individuals with tinnitus, a marked reduction in the amplitude of the waves, while the latencies are kept unaltered. This reduction in amplitude without changes in latency could be assigned to a reduction in the number of neurons responding to a reduction on neural activity and/or a larger mismatch of the firings of the neurons involved.

Another theory which can be considered to explain the mechanism responsible for the development of tinnitus is habituation, which is defined as a process of adaptation in the CNS which reduces or eliminates the perception of a stimulus introduced continuously and repeatedly. Thus, authors have suggested that individuals with frequent negative associations with tinnitus reinforce this perception and, as a consequence, do not get used to tinnitus, thus becoming chronic cases. Consequently, the high emotional background of severe tinnitus could lead to a high level of selective attention guided towards tinnitus, which could increase the lack of attention and/or prevent habituation from this symptom. Studies have reported that an attention-filled process can be involved in the delay or prevention of a habituation mechanism^4^^,^^5^.

The specialized literature shows that alterations in the central auditory tests and electrophysiological abnormalities in the Long Latency Auditory Evoked Potential (LLAEP) ^3^^,^^6^^,^^7^ may be found in individuals with tinnitus. Walpurger et al.^8^, studied habituation by means of the LLAEP in individuals with tinnitus using a series of auditory stimuli by means of four consecutive tests, using tone pips, reported that the results obtained were in accordance with the habituation theory, suggesting that patients with severe tinnitus fail in properly habituating to the sound stimulus.

Therefore, the goals of the present study were to characterize the results from long latency auditory evoked potentials (LLAEP), comparing the results obtained from these potentials from individuals with and without tinnitus, and to check the existence of association between the side with LLAEP alteration and the tinnitus location in individuals complaining of tinnitus exposed to high sound pressure levels in their jobs.

## MATERIALS AND METHODS

The present study was designed as a contemporary cross-sectional cohort, done in the Laboratory of Speech and Hearing Investigations in Auditory Evoked Potentials at the Speech and Hearing Therapy program of the Medical School of the University of São Paulo (FMUSP), approved by the Ethics Committee of the University of São Paulo Hospital, as well as by the Ethics Committee for Research Projects Analysis of the University of São Paulo Medical School Hospital - CAPPesq, under protocols # 712/06 and # 1278/06, respectively.

Such research protocols were based on Resolutions 196/96 and 251/97, from the National Health Board and Department of Health (CNS/MS), and the Free and Informed Consent Form had to be signed by the patient, consenting with the tests and use of the information obtained by the investigator. The material collected was stored in the institution's database.

The procedures were carried out only after the individuals signed the Free and Informed Consent Form.

The material from the present study was based on the results from the basic audiological evaluation and the Long Latency Auditory Evoked Potentials (latencies of components N1, P2 and P300 and N1-P2 amplitude) from 60 individuals exposed to occupational noise with high sound pressure levels (higher than 85 dBH), both from males and females, in the age range between 27 and 50 years of age, 30 with tinnitus (study group) and 30 without tinnitus (control group).

The selection of individuals complied with the following inclusion criteria: constant or intermittent tinnitus, uni or bilateral (experimental group); exposure to occupational noise with high sound pressure levels (higher than 85 dBH), and the noise level measurement was carried out by the Hygiene Division of Work Safety and Medicine Department of the University of São Paulo ((DHSMT USP); auditory thresholds within normal levels, in other words, lower than or equal to 25 dBHL in all the frequencies (0.25 kHz to 8 kHz) in both ears; Speech Recognition Threshold (SRT) equal to or up to 10 dB HL above the tritone average (500, 1K and 2K Hz)^9^, Percentage Index of Speech Recognition (PISR) between 90 and 100%(10); Type “A” tympanometric curve (pressure up to −100 daPa and volume between 0.3 and 1.6 cc)^11^; presence of contralateral stapes muscle acoustic reflex in the frequencies of 0.5kHz; 1kHz and 2kHz, based on Gelfand^12^.

We took off the study those individuals complaining of neurological, psychiatric and behavioral dysfunctions, and this data was obtained during the medical interview and the medical chart of the worker.

In order to select the subject to participate in the study, meeting the previously established inclusion criteria, we carried out the following procedures: interview; a questionnaire about tinnitus characteristics^13^, translated and adapted by Branco^14^, in order to collect data regarding localization, frequency, intensity and the factors which worsen tinnitus, among others; external acoustic meatus inspection through the use of a Heine otoscope; threshold tonal audiometry in the frequency range of 0.25 to 8 kHz by air transmission and, 0.5 to 4 kHz by bone transmission (in the frequencies with thresholds higher than 20dBHL buy air conduction) bilaterally; vocal audiometry (SRT and PISR), for that we used the GSI 61 and GSI 68 Grason-Stadler audiometers15; acoustic immittance measures (tympanometry with a 226Hz probe tone, and ipsilateral and contralateral acoustic reflexes from the stapes muscle in the frequencies of 0.5; 1 and 2 kHz^12^) using the GSI 33 middle ear analyzer from Grason-Stadler^16^.

After audiological evaluation, the selected individuals were submitted to electrophysiological evaluation of hearing (LLAEP) in order to obtain the latencies from components N1, P2, Cognitive Potential (P300) and N1-P2 latency. The individuals were placed in a reclining chair, in a poorly lighted room and were instructed to keep their eyes closed during the test, however being alert so as to perform the required cognitive activities. In order to carry out with this evaluation, we used the Traveler Express portable system from Bio-Logic, with the EP317 software^17^. The acoustic stimulus was introduced in a single ear by means of supra-aural phones (TDH-39), provoking responses both in the right and the left ears, and only one register was collected from each side and the registers from both sides were analyzed. The electrodes were placed on the right and left ears (A2 and A1), vertex (Cz) and forehead (Fpz), according to norm IES 10-20 (International Electrode System). We checked the values of electrode impedance, which should be below 5 kOhms. The acoustic stimulus used was the tone-burst at 75 dB HL, in the frequencies of 1 kHz (frequent stimulus) and 1.5 kHz (rare stimulus), randomly introduced by the computer. The rare stimulus happened between 15 and 20% from the total of 300 stimuli. The individual was instructed to keep his attention focused on the rare stimuli which randomly appeared among a series of frequent stimuli, and they were asked to count out loud the number of times the rare event happened^18^. We checked the presence and absence of these potentials, as well as their latency and amplitude when present.

All the tests were carried out in the morning, before the individual went to work, thus preventing the individual from being tired at the time of the test and guaranteeing that he/she would keep the same attention during LLAEP recording.

The results from the latency of components N1, P2 and P300 were initially classified in normal or altered, according to the normality criteria proposed by Williams et al.^19^ and McPherson^20^ for each age range, and are described on Chart 1, Chart 2, respectively, and later on the types of alterations found were described.

**Chart 1:** Normality pattern of the latency values (in ms) for waves N1 and P2, proposed by Williams et al. (1962)^19^

	N1 wave latency	P2 wave latency
Minimum	90 ms	160 ms
Maximum	150 ms	200 ms

**Chart 2:** Normality pattern for latency values (in ms) of the P300 wave for each age range, proposed by McPherson (1996)^20^

Ages	P300 wave latency Minimum	P300 wave latency Maximum
17 to 30 years	225 ms	365 ms
31 to 50 years	290 ms	380 ms

In analyzing results from the N1-P2 amplitude we only characterized the values obtained for not having in the literature a normality criterion established for this parameter.

The results from the electrophysiological evaluation, both in the study group and in the control group, who did not match the normality criteria aforementioned, were classified as altered. The individual was deemed altered when at least one of the ears or one of sides presented some alteration.

The altered latency results found from waves N1, P2 and P300 were classified in: “increase”, as to wave latency, it was increased if compared to the normal values; “absent”, when no wave was found; “both” when increase and absent alterations were found in the same individual.

After analyzing the scores assigned to tinnitus severity, the individuals were placed in three subgroups: group 1 - mild tinnitus; group 2 - moderate tinnitus; group 3 -severe tinnitus. Tinnitus severity was analyzed by means of the visual-analogue scale. According with such method, the individual was asked to provide a score from 1 to 10 to his tinnitus, considering that 1 would be mild tinnitus and 10 the worst tinnitus imaginable. The scores were then classified as: from 1 to 3 - mild tinnitus; from 4 to 6 - moderate tinnitus, and from 7 to 10 - severe tinnitus.

We used the Wilcoxon, Mann-Whitney, and Equality between two proportions and Chi-Squared tests. The values from the statistical analysis were considered statistically significant when p£0.05 and, all the confidence intervals were built with 95% statistical confidence.

## RESULTS

In the present study, the study group (SG) and the control group (CG) were made up of 30 individuals each, four women (13.33%) and 26 men (86.67%).

The age of the individuals in the study varied between 27 and 50 years with a mean of 41 years, and in the control group it varied between 27 and 50 years, with a mean of 41.6. We did not find statistically significant differences between the groups in relation to the mean age (p = 0.563).

**Figure 1:**
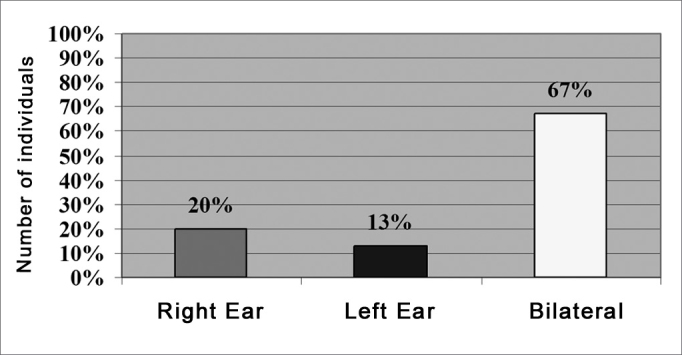
Results obtained in relation to tinnitus localization in the study group individuals (N=30).

Following, we will present the results obtained in relation to the tinnitus localization in the individuals from the study group (n=30). The data was evaluated as to laterality to the right ear (RE), left ear (LE), or both ears (BE). We noticed that of those individuals complaining of tinnitus, 66.7% had bilateral tinnitus, observing a statistically significant difference as to tinnitus location in the left and right ears (Fig. 1).

**Figure 2:**
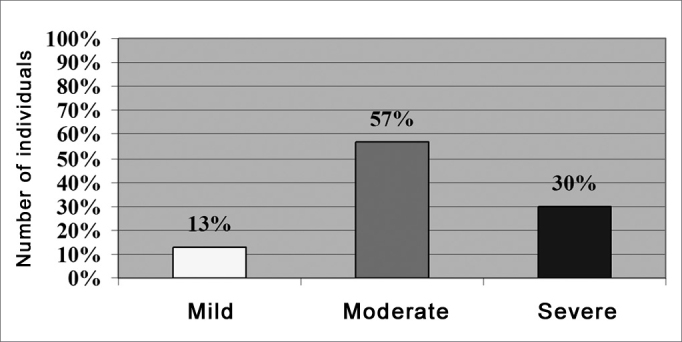
Results obtained in relation to the score mean values assigned to the tinnitus severity variable in the study group (N=30).

As far as tinnitus severity in individuals from the study group (n=30), the data was evaluated as to the level of discomfort caused by tinnitus into: mild, moderate and severe. We noticed that 56.7% of the individuals complaining of tinnitus had it in a moderate level, and we can see a statistically significant difference as to the mild (p < 0.001) and severe levels (p = 0.037) (Fig. 2).

The quantitative results obtained from comparing the mean latency values from components N1, P2, P300 (in ms) and the N1-P2 amplitude (in μV) between the right and left ears, in the control and study groups, which are depicted on Table 1. Since we did not find statistically significant differences between the right and left ears for both groups, the results were grouped and we compared these mean values between the control and study groups (Table 2). We noticed statistically significant differences between the control and study groups regarding the latencies of components N1, P2 and P300, and the results obtained from the study group were higher than those obtained from the control group.

**Table 1:** Comparison of the mean latency values of the N1, P2, P300 (in ms) components latency and N1-P2 amplitude (in μv), between the right and left ears in the control and study groups.

			Mean	Standard deviation	p-value
	N1	RE	90,8	12,7	0,583
		LE	90,7	11,8	
	P2	RE	187,1	26,1	0,076#
Control		LE	177,8	23,1	
	N1-P2	RE	8,34	2,57	0,067#
		LE	7,93	2,76	
	P300	RE	316,8	27,7	0,325
		LE	315,2	31,4	
	N1	RE	142,8	57,4	0,284
		LE	142,2	58,6	
	P2	RE	195,5	43,1	0,846
Study		LE	196,7	42,0	
	N1-P2	RE	9,53	5,51	0,393
		LE	9,43	5,13	
	P300	RE	326,4	45,1	0,088#
		LE	337,0	42,9	

p-value # near the threshold of acceptance, tending to be significant RE - right ear LE - left ear N - number of ears ms - milliseconds μv - microvolts

**Table 2:** Comparison of the mean values of the N1, P2, P300 (in ms) components of LLAEP and N1-P2 (in μv) amplitude between the study and control groups (N=60).

N1, P2 and P300	N1		P2		N1-P2		P300	
	CG	SG	CG	SG	CG	SG	CG	SG
Mean	90,8	142,5	182,5	196,1	8,14	9,48	316	331,7
Median	90	153	186	205	7,84	8,07	317	331
Standard deviation	12,2	57,5	24,9	42,2	2,65	5,27	29,4	44,0
p-value	<0,001*		0,002*		0,392		0,039*	

N - number of ears ms - milliseconds μv - microvolts

CG - Control Group SG - Study Group

* p-value considered statistically significant

Later on, we carried out a qualitative analysis of the results obtained from the LLAEP. For each potential we analyzed the occurrence of normal and altered results, as well as the types of alterations found in the study and control groups.

We observed statistically significant differences between the study and control groups as to the occurrence of normal and altered results regarding the latencies of components N1, P2 and P300 in LLAEP, respectively (Table 3). Within the control group we noticed a higher percentage of normal results (component N1), while in the study group there was a greater percentage of altered results (component P2). In both groups we observed a higher percentage of normal results in the P300 (Table 3). Having seen that the responses were present in all the individuals evaluated, and we did not observe alterations of the response absence type and both in the control and study groups. We must stress that all the alterations found in the LLAEP were of the increased latency type.

**Table 3:** Distribution of the occurrence of normal and altered results for the latency of LLAEP N1, P2 and P300 components, in the control and study groups.

	N1		P2		P300	
	GC	GP	GC	GP	GC	GP
Normal	100%	40%	73,3%	33,3%	96,7%	80%
Altered	0%	60%	26,7%	66,7%	13,3%	20%
p-value	<0,001*		0,002*		0,044*	

CG - Control Group

SG - Study Group

* p-value considered statistically significant

The study of the association between the side harboring the alteration in auditory evoked potentials and tinnitus location was carried out in the study group, yielding a deeper association between the side of the N1, P2 and P300 components' alterations and tinnitus location, especially when the tinnitus is bilateral, although we did not see statistically significant differences regarding this association (Table 4).

**Table 4:** Association between the side of the alteration in LLAEP N1, P2 and P300 components and the tinnitus localization in the study group (N=30).

		RE		LE		Bilateral		Total	
	N1	N	%	N	%	N	%	N	%
	RE (N=6)	1	33%	1	33%	1	34%	3	100%
Tinnitus	LE (N=4)	0	0%	0	0%	0	0%	0	0%
	Bilateral (N=20) P2	3	20%	1	7%	11	73%	15	100%
	RE (N=6)	0	0%	1	50%	1	50%	2	100%
Tinnitus	LE (N=4)	1	33%	0	0%	2	67%	3	100%
	Bilateral (N=20) P300	1	7%	3	20%	11	73%	15	100%
	RE (N=6)	0	0%	0	0%	0	0%	0	100%
	LE (N=4)	0	0%	0	0%	0	0%	0	100%
	Bilateral (N=20)	1	17%	2	33%	3	50%	6	100%

N - number of individuals

RE - Right Ear

LE - Left Ear

p-value for N1 = 0.301 p-value for P2 = 0.456.

## DISCUSSION

The long latency auditory evoked potentials (LLAEP), also known as late potentials or event-related potentials, were first studied by Picton et al.^21^, which named them according to their polarity (negative/positive) and latency.

In the present study, we analyzed the N1, P2, P300 components from the LLAEP. Wave N1 presents as possible generators the primary auditory cortex (upper portion of the temporal lobe), which can be associated to the attention given to the sound stimulus, and secondary auditory cortex (lateral-most portion of the temporal lobe)^22^. The P2 wave does not present its generators so well established; nonetheless, different areas from the primary and secondary auditory cortex, as well as the reticular formation^23^, seem to be responsible for the generation of such component. By the same token, it is believed that the P300 has the frontal cortex, the centro-parietal cortex and the hippocampus as generators^20^.

Initially, we compared the mean latency values of components N1, P2, P300 and N1-P2 amplitude between the study and control groups. We also noticed that the study group presented higher mean values in all the components assessed when compared to the control group, with statistically significant differences for latencies from waves N1 (p<0.001), P2 (p=0.002) and P300 (p=0.039), except for N1-P2 amplitude (p=0.392) (Table 2).

As it happened in the present study, Jacobson et al.^4^ found higher mean values in the group with tinnitus for components: N1 (103.13 - GC and 119.73 - GP). However, component P2 (209.01 - GC and 205.80- GP) had such which were higher in the control group.

Despite the lack of a significant difference for N1-P2 amplitude in the comparison between the study and control groups (Table 2), we noticed higher mean amplitude values for the tinnitus group. This finding can be explained by the habituation theory. In the study from Walpurger et al.^8^ they noticed, by means of the LLAEP, a lower habituation of the N1-P2 amplitude difference in individuals with tinnitus when compared to individuals without tinnitus, suggesting that patients with severe tinnitus fail in properly habituating regarding repeated and meaningless sounds.

In the literature studied, we found some studies which used different types of LLAEP to assess individuals with tinnitus, and these found latency and/or amplitude alterations in the potentials^3^^,^^8^^,^^22^^,^^24^.

In the quantitative analysis of normal and altered result occurrence distribution regarding N1, P2 and P300 components latency from the LLAEP, in the control and study group, it was noticed that the study group had a higher percentage of altered results (60%, 66.7% e 20%, respectively) when compared to the control group, which presented 100%, 73.3% and 96.7% of normal results, respectively (Table 3). When comparing the occurrence of normal and altered results for P2 and P300 components, such difference was shown for both groups (Table 3).

The large number of altered results seen in components N1 and P2 in individuals with tinnitus suggest that the presence of alterations in different areas of the primary auditory cortex.^22^^,^^23^.

Considering the types of alterations present in components N1, P2 and P300 we noticed that the only type of alterations present, for both groups, was the increase in N1, P2 and P 300 component latencies.

These findings corroborate those from Jacobson et al.^4^, who reported that the latency increase in components N1 and P2 from the LLAEP, was the most commonly found alteration in patients with tinnitus. On the other hand, Attias et al.^3^, studied LLAEP in individuals with tinnitus and observed that the latencies from components N1, P2 and P300 were kept unaltered; nonetheless showed a marked reduction in the amplitude of these components. Other authors have also reported on the reduction of N1^8^^,^^24^, P2^8^ and P300^25^ amplitudes in the group with tinnitus.

Picton (1992)^26^ reported that the increase in latency or reduction in amplitude in the LLAEP is associated with clinical and subclinical problems. Having said that, it believed that a deficit in some central auditory processing skill, with a reduction in the auditory attention^4^^,^^5^^,^^14^^,^^27^, memory deficit^28^^,^^29^, difficulties in frequency discrimination and sound intensity with good temporal and/or binaural interaction^30^ can cause the changes on the characteristics of the LLAEP components in individuals with tinnitus.

Other possible factors which can be associated to the changes seen in the LLAEP in individuals with tinnitus are: the possibility of reduction in the number of working neurons, reduction in neural activity and/or a greater mismatch of the firings of the involved neurons^3^.

Very little is known about the electrophysiological characteristics of individuals with tinnitus, as well as on the interaction of the attention and tinnitus mechanism^4^.

According with Coelho et al.^31^, patients with tinnitus frequently complain of concentration difficulties in daily activities, which could be higher or lower, according to the attention given to this symptom. It is known that components N1, P2, P300 are influenced by the degree of attention given to the stimulus. If the stimulus is ignored, the wave shapes are damped and, very likely delayed^18^. Another factor which must be taken into account is the fact that tinnitus has a masking effect on the acoustic signals presented to these individuals^14^. Thus, one could infer on the hypothesis that the less attentive individuals from the study group, very likely due to the presence of tinnitus, could have this reduced attention as a contributing factor for a LLAEP latency increase.

Because of this analysis we notice that the LLAEP alterations seen in individuals with tinnitus show an involvement of the CANS, suggesting a participation of the auditory cortex in the generation and/or tinnitus maintenance. Thus, the LLAEP is a useful tool to investigate the mechanism responsible for this symptom.

In studying the association between the altered side of the auditory evoked potentials and tinnitus location, we noticed a greater association between the bilateral tinnitus and the bilateral alteration in N1, P2 and P300 LLAEP components, and the differences were not significant (Table 4).

We did not find in the literature studies associating the side of tinnitus with the side of the LLAEP alteration in individuals with hearing thresholds within normal values, complaining of tinnitus exposed to high sound pressure levels, thus making it difficult to compare the results from the present paper with those from other studies. The fact that this study showed a positive association between tinnitus localization and LLAEP alteration provides clues as to CNAS in the generation of tinnitus.

## CONCLUSION

We noticed a greater occurrence of LLAEP alterations in individuals with tinnitus when compared to individuals without tinnitus. The most commonly found type of alteration in this population was the increase in latency values, suggesting the existence of a possible CNAS dysfunction at a cortical level in individuals with tinnitus exposed to occupational noise. We also noticed a greater association between the side of LLAEP N1, P2 and P300 component alteration and the tinnitus localization when the individuals had bilateral tinnitus.
